
JOURNAL INFORMATION
==============================
NLM Title Abbreviation: Wirtschaftsdienst
Journal ID: Wirtschaftsdienst
NLM Journal ID: 101086612

Wirtschaftsdienst (Hamburg, Germany : 1949)

ISSN: 0043-6275

ARTICLE INFORMATION
==============================
PMCID: PMC7964467
PMID: 33746299
DOI: 10.1007/s10273-021-2862-0
Article ID: 2862
Article version: 1
Subjects: Kurz Kommentiert

Weiterbildungskonzept für Krisen

Weber Enzo Enzo.Weber@iab.de

Forschungsbereich A1, Inst. f. Arbeitsmarkt- und Berufsforschung, Regensburger Str. 100, 90478 Nürnberg, Deutschland
Electronic publication date: 2021 Jun 23
Print publication date: 2021
Volume: 101
Issue: 3
First page: 154
Last page: 154
Copyright: © Der/die Autor:in(nen) 2021
License: Open Access: Dieser Artikel wird unter der Creative Commons Namensnennung 4.0 International Lizenz veröffentlicht (https://creativecommons.org/licenses/by/4.0/deed.de).
Open Access wird durch die ZBW — Leibniz-Informationszentrum Wirtschaft gefördert.
License URL: https://creativecommons.org/licenses/by/4.0/

issue-copyright-statement: © The Author(s) 2021

==============================

Rezessionen sind die beste Zeit für Weiterbildung: Die Auslastung der Betriebe sinkt, Arbeitskapazität bleibt frei. Und Rezessionen sind zugleich Zeiten großer Umbrüche. Der strukturelle und technologische Wandel entlädt sich immer dann am Arbeitsmarkt, wenn die kontinuierliche Entwicklung ohnehin erschüttert wird. Somit ist auch der Bedarf, Kompetenzen weiterzuentwickeln, besonders groß. Gerade die Corona-Krise ist eine transformative Rezession (Hutter und Weber, 2020), die den digitalen und ökologischen Wandel noch verstärkt.

Dennoch haben in der Corona-Krise gerade einmal 5 % der Kurzarbeitenden die ausgefallene Zeit für Weiterbildung genutzt (Kruppe und Osiander, 2020). Dabei würde sich die Qualifizierung durchaus lohnen: Durch positive Lohn- und Beschäftigungseffekte ließe sich über höhere Steuern und Beiträge sogar ein erheblicher Teil der Kosten der Kurzarbeit für die öffentliche Hand zurückgewinnen (Kruppe et al., 2020). Die geringe Beteiligung dürfte nicht nur durch die Infektionslage begründet sein, denn schon in der Rezession 2009 war das gleiche dürftige Ergebnis zu beobachten. Nach den Gründen gefragt, geben Betriebe hauptsächlich die Unsicherheit der weiteren Geschäftsentwicklung und Probleme bei der Anpassung der Weiterbildung im Arbeitsplan an (Bellmann et al., 2020). Darauf müssen wir bei der nächsten Krise vorbereitet sein. Qualifizierung will normalerweise wohlgeplant sein – zeitliche Abläufe, strategische Ziele, Personalentwicklung. Aber besonders wichtig wäre sie offenbar in Situationen, die kaum planbar sind. Dafür wären vor allem kurzfristig und flexibel einsetzbare Weiterbildungsangebote entscheidend. Digitale Komponenten dürften dabei – auch ohne Kontaktverbote – eine wichtige Rolle spielen, ebenso wie ein inhaltlich und organisatorisch modularer Aufbau. Derartige Konzepte zu entwickeln, ist auch Aufgabe der Weiterbildungsbranche – die von der Politik dafür entsprechend gefördert werden sollte. Denn das Vorhalten solcher Konzepte, die erst zu einem ungewissen Zeitpunkt in der Zukunft nachgefragt werden, ist ein Beitrag zur Krisenreaktionsfähigkeit der gesamten Volkswirtschaft.

Zu diesem Konzept gehören auch geeignete Anreize. So könnten Verlängerungsmöglichkeiten des Kurzarbeitergelds oder die Übernahme der Sozialversicherungsbeiträge an Qualifizierungsbemühungen geknüpft werden. Man sollte dagegen auch nicht gelten lassen, dass eine solche Qualifizierung für viele Ungelernten-Jobs überhaupt nicht notwendig wäre; die Perspektive muss über die aktuell ausgeübte Tätigkeit hinaus gehen, die sich ohnehin durch den Wandel des Arbeitsmarkts schnell ändern kann.

Hält man für die Qualifizierungsbemühungen die Anforderungen hoch, sollten die Möglichkeiten zur Erfüllung der Anforderungen für die ohnehin mit einem Wirtschaftseinbruch befassten Betriebe aber möglichst flexibel gestaltet werden (Weber, 2020). So wäre verpflichtend, etwa nur einen Teil des Arbeitsausfalls mit Qualifizierung zu füllen; Mindestdauern einzelner Maßnahmen sollten nicht verlangt werden; und in begrenztem Umfang könnten Inhalte anerkannt werden, die neben Fertigkeiten für den allgemeinen Arbeitsmarkt auch stärker betriebsspezifische Kompetenzen betreffen. Entsprechend sollten die Voraussetzungen für die öffentliche Förderung von Weiterbildung ausgestaltet werden, hier liegen die Anforderungen gemessen an Krisensituationen derzeit noch recht hoch.

Weiterhin sollte es sowohl für Betriebe als auch Beschäftigte professionelle Hilfestellung geben. Es kommt also besonders darauf an, Qualifizierungsberatung bereitzustellen, die auch in Ausnahmesituationen Informationen und Anregungen zu Weiterbildungsorganisation, -formaten und -inhalten vermitteln kann. Schließlich ist auch an Anreize für die Beschäftigten zu denken. So könnte bei Teilnahme an Weiterbildung eine anrechnungsfreie Prämie zusätzlich zum Kurzarbeitergeld bezahlt werden. Entsprechend sollte in der Arbeitslosigkeit ein Bildungsbonus über die passiven Leistungen hinaus eingeführt werden. All diese Komponenten würden ein Weiterbildungskonzept für Rezessionen ausmachen. Vor dem Hintergrund der aktuellen und der vergangenen Krise sollte dieses im Vorhinein ausgearbeitet werden, sodass wir beim nächsten Mal schnell, transparent und flexibel reagieren können. Wir müssen Qualifizierung effektiver umsetzen, wenn es darauf ankommt.

Enzo Weber
Institut für Arbeitsmarkt- und Berufsforschung (IAB)
enzo.weber@iab.de


Literatur

Hutter C Weber E Corona-Krise: die transformative Rezession Wirtschaftsdienst 2020 100 6 429 431 10.1007/s10273-020-2676-5 32834158 PMC7335928
Kruppe, T. und C. Osiander (2020), Kurzarbeit im Juni 2020: Rückgang auf sehr hohem Niveau, IAB-Forum, 23.9.
Kruppe, T., E. Weber und J. Wiemers (2020), Qualifizierung senkt die Nettokosten der Kurzarbeit, IAB-Forum, 24.8.
Bellmann, L., P. Gleiser, C. Kagerl, E. Kleifgen, T. Koch, T. Kruppe, C. König, J. Lang, U. Leber, L. Pohlan, D. Roth, M. Schierholz, J. Stegmaier und A. Aminian (2020), Weiterbildung in der Covid-19-Pandemie stellt viele Betriebe vor Schwierigkeiten, IAB-Forum, 9.12.
Weber, E. (2020), Kurzarbeit in der Corona-Krise: Längere Bezugsdauer bei Qualifizierung der Beschäftigten, IAB-Forum, 6.7.
